# Mitochondrial Displacement Loop Region SNPs Modify Sjögren’s Syndrome Development by Regulating Cytokines Expression in Female Patients

**DOI:** 10.3389/fgene.2022.847521

**Published:** 2022-03-11

**Authors:** Yufei Zhao, Chenxing Peng, Jingjing Zhang, Ruixue Lai, Xiaoyun Zhang, Zhanjun Guo

**Affiliations:** ^1^ Department of Immunology and Rheumatology, The Fourth Hospital of Hebei Medical University, Shijiazhuang, China; ^2^ Department of Immunology and Rheumatology, The Second Hospital of Hebei Medical University, Shijiazhuang, China

**Keywords:** Sjögren's syndrome, D-loop, snps, MtDNA copy number, ROS, cytokine

## Abstract

Mitochondrial dysfunction could induce innate immune response with cytokines releasing to initiate Sjögren’s syndrome (SS) onset. Single nucleotide polymorphisms (SNPs) in the mitochondrial displacement loop (D-loop) and mitochondrial DNA (mtDNA) copy number of female SS patients were evaluated for their association with SS in female patients. At the nucleotide site of 152, 16304, 16311 and 16362 in the D-loop, the frequencies for the minor alleles of 152C (*p* = 0.040, odds ratio [OR] = 0.504), 16304C (*p* = 0.045, OR = 0.406), 16311C (*p* = 0.045, OR = 0.406) and 16362C (*p* = 0.028, OR = 0.519) were significantly higher in the SS patients than those in the female controls, which indicated that 152,C, 16304C, 16311C, and 16362C allele in the D-loop of mtDNA were associated with the risk of SS. Meanwhile, the excessive SNPs were accumulated in D-loop region of SS patients (8.955 ± 2.028 versus 7.898 ± 1.987, *p* < 0.001, 95% confidence interval [CI]: 0.477–1.637) and mtDNA copy number increased in SS patients (1.509 ± 0.836 versus 1.221 ± 0.506, *p* = 0.006, 95% CI: 0.086–0.490) by a case-control analysis. The subsequent analysis showed that SS risk-related allele 16311C was associated with higher IL-2 levels (*p* = 0.010) at significantly statistical level whereas 152C associated with lower IL-10 levels (*p* = 0.058) at a borderline statistical levels. Our findings suggest that mitochondrial D-loop SNPs are predictors for SS risk, it might modify the SS development by regulating cytokine expression.

## Introduction

Sjögren’s syndrome (SS) is a chronic autoimmune disease characterized by hypofunction of the exocrine glands (particularly the lacrimal and salivary glands), which results in dry eyes and mouth symptoms. The estimated prevalence is 0.5–1.5% with a female to male ratio of 9:1 worldwidely (Qin et al., 2015). The etiology of SS including genetic susceptibility, epigenetic alteration, infectious agents, oxidative stress, cytokines (Roescher et al., 2009; Wakamatsu et al., 2013; Bartoloni et al., 2019), but the real formation mechanism for SS is not clear.

Human mitochondrial DNA (mtDNA), a 16 kb circular double-stranded DNA molecule, is more prone to mutations than nuclear DNA due to its inefficient repair system, frequent exposure to ROS, and the lack of protective histones (Gonçalves, 2019; Sun et al., 2019). Both qualitative (mutations and polymorphisms) and quantitative (mtDNA copy number) alterations in mtDNA initiates the onset of many illnesses (Warowicka et al., 2013). The displacement loop (D-loop) is an unique non-coding mtDNA region that controls both mitochondrial genome replication and expression (Nguyen et al., 2020). Single nucleotide polymorphisms (SNPs) in this region might induce mitochondrial dysfunction thereby to initiate ROS overproduction, aberrant energy expenditure, and auto-antigen production (Frezza, 2014). SNPs in the D-loop have been shown to associated with a range of immune rheumatism including rheumatoid arthritis, and systemic lupus erythematosus (Zhang et al., 2017; Lai et al., 2020). The mtDNA copy number is tightly regulated to ensure that mitochondria are able to produce appropriate amount of energy and intracellular signals to maintain normal cellular functions (Zhang et al., 2017). MtDNA copy number, which reflects gene-environment interactions between unknown genetic variables and oxidative stress exposures, has been shown to be a risk predictor for breast cancer, cardiovascular illness, and neurological disease (Shen et al., 2010; Eirin et al., 2016; Pyle et al., 2016). MtDNA changes could trigger the innate immune response and cause the release of downstream cytokines, whose dynamic balance is essential for maintaining the homeostasis of the immune system (Liu et al., 2013; Barrera et al., 2021). The imbalance of pro-inflammatory and anti-inflammatory cytokines could do damage to secretory functions of gland in SS patients (Roescher et al., 2009). In addition, abnormal ROS levels have been shown to be responsible for cellular dysfunction or autoimmune responses in the development of SS (Kowaltowski and Vercesi, 1999).

We performed a mtDNA-based assessment to evaluate its association with the development of SS, we also focused on ROS and cytokine involvement in SS patients. In order to exclude the influence of sex hormones, we only used female patients for related research.

## Materials and Methods

### Tissue Specimens and DNA Extraction

Blood samples were taken from 89 female SS patients from the Department of Immunology and Rheumatology at The Second Hospital of Hebei Medical University between June 2021 and October 2021. SS was diagnosed using the 2016 American College of Rheumatology/European League Against Rheumatism (ACR-EULAR) classification criteria (Shiboski et al., 2017). We collected the clinical characteristics including age, dry eye, dry mouth, rampant caries, parotid gland enlargement, arthritis, renal tubule acidosis, interstitial lung disease (ILD), leukopenia, thrombocytopenia, and some laboratory tests such as antinuclear antibody (ANA), Anti-SSA, Anti-SSB, ESSDAI, ESR, CRP. At the same time, 98 female healthy controls who had no history of autoimmune diseases were also included in the study. The serum sample was venous blood collected from patients fasted overnight. DNA was extracted with the Genomic DNA extraction kit (Tiangen, Beijing, China) using blood samples. The Second Hospital of Hebei Medical University’s Human Tissue Research Committee approved all procedures. Informed consent was obtained prior to enrollment from every participant.

### Polymerase Chain Reaction Amplification and Sequence Analysis

The primers used in this study were 5′-CCC​CAT​GCT​TAC​AAG​CAA​GT-3′ (nucleotides 16190–16209) and reverse 5′-GCT​TTG​AGG​AGG​TAA​GCT​AC-3′ (nucleotides 602–583) for amplification of a 982 bp product in the D-loop. DreamTaq Green PCR Master Mix Kit (2×) (Thermo Fisher Scientific, Waltham, MA, United States) was used for PCR and purification before sequencing. The BigDye Terminator v3.1 Cycle Sequencing Kit (Life Technologies, Carlsbad, CA, United States) was used to carry out cycle sequencing and the ABI PRISM Genetic Analyzer 3100 (Applied Biosystems) was used to separate the products. Polymorphisms were confirmed by repeated analysis of both strands. The Second Hospital of Hebei Medical University’s Human Tissue Research Committee approved all procedures.

### Measurement of mtDNA Copy Number

Quantitative real-time polymerase chain reaction (qRT-PCR) analyses were performed by an Applied Biosystems 7500 Sequence Detection System to measure the relative mtDNA copy number (Applied Biosystems, Foster City, CA). Human β-haemoglobin (HGB) and mitochondrial nicotinamide adenine dinucleotide (NADH) dehydrogenase 1 (ND1) genes were used to examine nuclear DNA and mitochondrial DNA, respectively (Xing et al., 2008).The primers were listed in Supplementary Table S1. The genomic DNA (30 ng) was mixed with 3µl of qPCR SYBR Green Mix (5×) (GeneCopoeia, Rockville, Md, United States) containing 10 pmol forward and reverse primers in a final volume of 15 μl. The amplification was done under the same conditions as previously described in the previous section (Zhan et al., 2020). Amplification specificity was determined by using melting curve analysis. The copy number of mtDNA in each specimen was estimated using the 2-ΔΔCt relative expression formula (ΔCt = Ct ND1-Ct HGB). Measurements were repeated twice with a sample of unchanged DNA in each well.

### ROS Measurement

Serum total ROS levels were determined using BBOXiProbe^®^ Plasma Active Oxygen Detection Kit (BestBio Technology, Shanghai, China). Briefly, 100μl serum was incubated for 30 min at 37°C with 10 μl O12 probe. We measured ROS levels using a Fluorescence Microplate Reader (BIOTEK, Winooski, VT, United States) with an excitation wavelength of 488 nm and an emission wavelength of 520 nm.

### Cytokines Measurement

Interleukin-5 (IL-5), Interleukin-13 (IL-13), interferon-γ (IFN-γ), Interleukin-2 (IL-2), Interleukin-6 (IL-6), Interleukin-10 (IL-10), Tumor Necrosis Factor-α (TNF-α), and Interleukin-4 (IL-4) were measured using the Human TH1/TH2 Panel (8-Plex) with Filter Plate V02 (Biolegend, San Diego, CA). 25µl serum samples (two-fold diluted with Assay Buffer) were incubated with 25µl antibody and mixed with beads for 1 h at room temperature, shaking at approximately 800 rpm. Then, 25µl of streptavidin-phycoerythrin (SA-PE) was added to each medium, which was shaken at approximately 800 rpm for 30 min at room temperature in the dark. Using the MACSQuant Analyzer 10 (Miltenyi Biotec, Bergisch Gladbach, Germany), the PE fluorescence signal of the analyte-specific bead region was quantified and the concentration of the analyte was determined by the LEGENDplexTM software from BioLegend (Biolegend, San Diego, CA).

### Statistical Analysis

For continuous variables, Student’s *t* test was used. Wilcoxon rank-sum test was used if the assumptions of normality for the *t*-tests were not met. For assessing the independence of categorical variables in contingency tables, the Chi-square test or Fisher’s exact test was used. As for assessing the relationship between variables, Pearson’s correlation test was used. If normality is not assumed, Spearman’s correlation test will be used. All statistical analyses were performed using SPSS software version 19.0 (IBM Corporation, Armonk, NY). *p* < 0.05 was considered to indicate statistical significance.

## Results

A total of 89 female subjects with SS and 98 gender-matched healthy controls were included in the study. The clinical features of the patients and controls of this study can be seen in Table 1, no statistical difference existed referring to Age.

**TABLE 1:** Clinical characteristics of SS patients and controls.

Group	SS patients (*n* = 89)	Controls (*n* = 98)	T value	*p*
Age (year)	49.66 ± 13.53	51.72 ± 10.85	−1.142	0.255
Manifestations				
Dry eye	75 (84.3%)			
Dry mouth	48 (53.9%)			
Rampant caries	39 (43.8%)			
Parotid gland enlargement	10 (11.2%)			
Arthritis	14 (15.7%)			
Renal tubule acidosis	17 (19.1%)			
ILD	24 (27.0%)			
Leukopenia	24 (27.0%)			
Thrombocytopenia	16 (18.0%)			
ANA (+)	80 (89.9%)			
Anti-SSA (+)	68 (76.4%)			
Anti-SSB (+)	40 (44.9%)			
ESSDAI				
<5(Low)	18 (20.2%)			
5 ≤ ESSDAI≤13(Moderate)	62 (69.7%)			
ESSDAI≥14(High)	9 (10.1%)			
ESR(mm/h)	42.99 ± 30.59			
CRP(mg/L)	12.49 ± 31.05			

SS, sjogren’s syndrome; *p*, probability value; ILD, interstitial lung disease; ANA, antinuclear antibody; ESSDAI, EULAR, Sjögren’s syndrome (SS) disease activity index.

**FIGURE 1:**
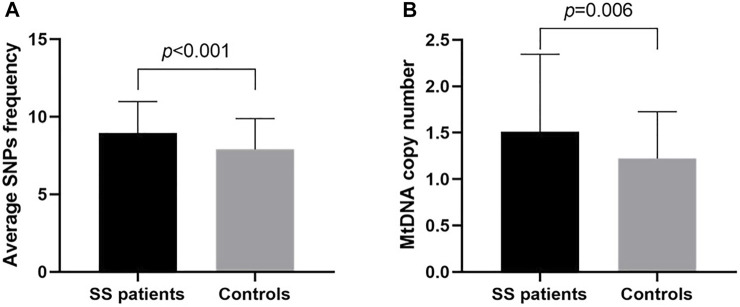
Average SNPs frequency, mtDNA copy number in SS patients and controls. **(A)** The average frequency of SNPs in each SS patient was significantly greater than in controls. **(B)** MtDNA copy number in SS patients was significantly higher than that of controls. SS, Sjogren’s syndrome; SNP, single nucleotide polymorphisms; MtDNA, mitochondrial DNA; *p*, probability value.

SNPs were identified in 100 sites in the mitochondrial D-loop of the SS patients. The average frequency of SNPs was significantly greater in PM/DM patient than that in controls (8.955 ± 2.028 versus 7.898 ± 1.987, *p* < 0.001, 95% confidence interval [CI]: 0.477–1.637, Figure 1A), which indicated that D-loop SNPs were accumulated in SS patients. 25 SNPs with minor alleles frequency higher than 5% in either SS patients or controls were used for risk analysis (Table 2). At the nucleotide site of 152, 16304, 16311 and 16362 in the D-loop, the frequencies for the minor alleles of 152C (*p* = 0.040, odds ratio [OR] = 0.504), 16304C (*p* = 0.045, OR = 0.406), 16311C (*p* = 0.045, OR = 0.406) and 16362C (*p* = 0.028, OR = 0.519) were significantly higher in the SS patients than those in the female controls, which indicated that 152, 16304, 16311, and 16362C alleles in the D-loop of mtDNA were associated with the risk of SS. Moreover, mtDNA copy number was significantly higher in SS patients than in controls (1.509 ± 0.836 versus 1.221 ± 0.506, *p* = 0.006, 95% CI: 0.086–0.490, Figure 1B). The relationship between mtDNA copy number and SS risk-associated SNPs was compared by t-tests subsequently, but no correlation was found (data not shown). The linkage disequilibrium analysis was also performed to analyze the interaction among SS risk-associated SNPs, which revealed no association among these SNPs (*r* ^*2*^ < 0.05).

**TABLE 2:** Single nucleotide polymorphism sites showing frequency difference between SS patients and controls.

**Nucleotide**	**SS**	**Controls (*n* = 98)**	**χ^2^**	***p***	**OR**	**95%CI**
	**patients**					
	**(*n* = 89)**					
73G/A	86/3 (96.6%/3.4%)	98/0 (100.0%/0%)	1.561	0.211	0.966	0.930–1.005
146T/C	77/12 (86.5%/13.5%)	84/14 (85.7%/14.3%)	0.025	0.874	1.069	0.466–2.454
150C/T	71/18 (79.8%/20.2%)	83/15 (84.7%/15.3%)	0.776	0.378	0.713	0.335–1.517
151C/T	84/5 (94.4%/5.6%)	93/5 (94.9%/5.1%)	0.000	1.000	0.903	0.253–3.230
152T/C	59/30 (66.3%/33.7%)	78/20 (79.6%/20.4%)	4.212	0.040	0.504	0.261–0.975
195T/C	78/11 (87.6%/12.4%)	90/8 (91.8%/8.2%)	0.090	0.343	0.630	0.241–1.646
199T/C	84/5 (94.4%/5.6%)	94/4 (95.9%/4.1%)	0.022	0.882	0.715	0.186–2.750
204T/C	83/6 (93.3%/6.7%)	95/3 (96.9%/3.1%)	1.379	0.240	0.437	0.106–1.802
207G/A	83/6 (93.3%/6.7%)	96/2 (98.0%/2.0%)	1.500	0.221	0.288	0.057–1.467
235A/G	79/10 (88.8%/11.2%)	89/9 (90.8%/9.2%)	0.215	0.643	0.799	0.309–2.066
249A/del	63/26 (59.6%/40.4%)	75/23 (76.5%/23.5%)	0.796	0.372	0.743	0.387–1.428
263A/G	87/2 (97.8%/2.2%)	96/2 (98.0%/2.0%)	0.000	1.000	0.906	0.125–6.572
309insertC^a^/C	87/2 (97.8%/2.2%)	97/1 (99.0%/1.0%)	0.007	0.933	0.448	0.040–5.032
489T/C	38/51 (42.7%/57.3%)	51/47 (52.0%/48.0%)	1.633	0.201	0.687	0.385–1.223
523-524AC/del	55/34 (61.8%/38.2%)	56/42 (57.1%/42.9%)	0.419	0.517	1.213	0.676–2.179
16260C/T	82/7 (92.1%/7.9%)	93/5 (94.9%/5.1%)	0.593	0.441	0.630	0.192–2.061
16261C/T	83/6 (93.3%/6.7%)	95/3 (96.9%/3.1%)	0.693	0.405	0.437	0.106–1.802
16266C/T	84/5 (94.4%/5.6%)	97/1 (99.0%/1.0%)	1.867	0.172	0.173	0.020–1.512
16290C/T	80/9 (89.9%/10.1%)	91/7 (92.9%/7.1%)	0.526	0.468	0.684	0.244–1.920
16298T/C	80/9 (89.9%/10.1%)	87/11 (88.8%/11.2%)	0.060	0.806	1.124	0.443–2.854
16304T/C	73/16 (82.0%/18.0%)	90/8 (91.8%/8.2%)	4.016	0.045	0.406	0.164–1.001
16311T/C	73/16 (82.0%/18.0%)	90/8 (91.8%/8.2%)	4.016	0.045	0.406	0.164–1.001
16319G/A	80/9 (89.9%/10.1%)	86/12 (87.8%/12.2%)	0.213	0.645	1.240	0.496–3.101
16362T/C	43/46 (48.3%/51.7%)	63/35 (64.3%/35.7%)	4.845	0.028	0.519	0.289–0.933
16519T/C	51/38 (57.3%/42.7%)	60/38 (61.2%/38.8%)	0.297	0.586	0.850	0.474–1.525

a Including C and CC, insertion.

SS, Sjogren’s syndrome; χ^2^:Chi-square; *p*, probability value; OR, odds ratio; CI, confidence interval.

Another 40 SS patients was used in an independent cohort to test the validation of our results, the alleles of 152C (*p* = 0.036, OR = 0.427), 16304C (*p* = 0.041, OR = 0.306), 16311C (*p* = 0.041, OR = 0.306), and 16362C (*p* = 0.037, OR = 0.455) did show their association with SS risk again (Supplementary Table S2), and mtDNA copy number in SS patients was also higher than that in controls (1.874 ± 1.049 versus 1.221 ± 0.506, *p* < 0.001, 95%CI: 0.304–1.002, Supplementary Figure S1).

**FIGURE 2:**
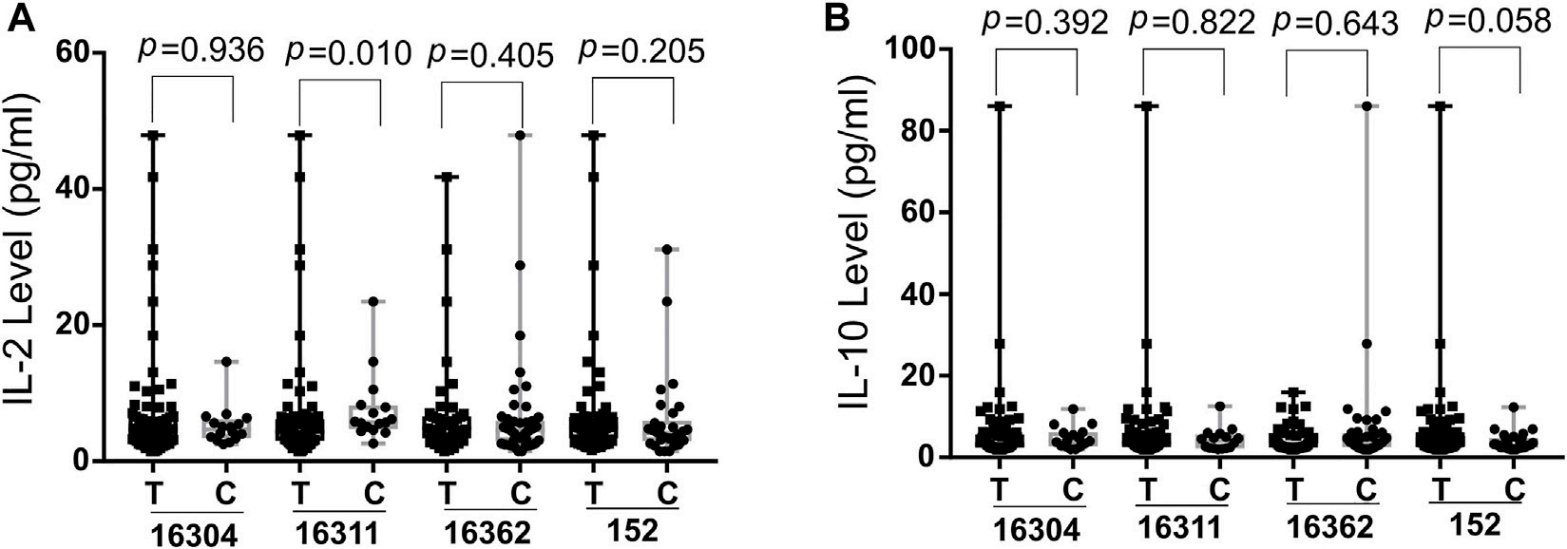
Boxplot of IL-2 **(A)** and IL-10 **(B)** levels in SS risk-associated SNPs. Wilcoxon rank sum test was used to determine significance IL-2, Interleukin-2; IL-10, Interleukin-10; SS, Sjogres syndrome; SNP, single nucleotide polymorphisms; p, probability value.

The potential correlation of cytokines (IL-5, IL-13, IFN-γ, IL-2, IL-6, IL-10, TNF-α and IL-4) levels and SS risk associated D-loop SNPs were evaluated using the Wilcoxon rank sum test (Figure 2, Supplementary Figure S2). As shown in Figure 2, 16311C was associated with higher IL-2 levels (*p* = 0.010) at significantly statistical level whereas 152C associated with lower IL-10 levels (*p* = 0.058) at a borderline statistical levels. These data implied that SS susceptible SNPs might modify SS development by mediating cytokine expression. The relationship between ROS levels and SS risk-associated SNPs was also explored with blood samples of patients, but no significant associations were found (data not shown).

## Discussion

Mitochondria play essential roles in regulating reactive oxygen species (ROS) signaling, energy production, calcium homeostasis, and cell apoptosis. As a non-coding portion of mtDNA, the D-loop region is highly associated with the complete mtDNA replication and transcription processes. Some severe changes in this area may disrupt the stability of mitochondrial activity to change in immunological status (Malik et al., 2015; Nguyen et al., 2020). We found mtDNA changes inducing D-loop SNPs and copynumber were associated with SS risk. Our samples are enough for detecting the difference of SNPs distribution frequency in the SS patients based on PASS 15 analysis. The hypervariable (HV) segment region of the D-loop has been identified as a somatic mutational “hotspot” in a number of diseases (Hibi et al., 2001), all of the SS-risk related SNPs we have identified is located in this region (16304, 16311 and 16362 in HV-I, 152 in HV-II). Based on our previous studies, the 16304 allele associated with survival of Non-Hodgkin lymphoma, whereas 16311 and 16362 associated with age-related onset for both familial breast and non-small cell lung carcinoma (Diao et al., 2015; Hu et al., 2015; Lee et al., 2016). The 152C allele was also shown to be substantially related to metastasis of malignant fibrous histiocytoma (Luo et al., 2019). The phenomenon that all susceptible loci of SS are concentrated in this region only reflects the high mutagenicity here or implied the predisposing factors of immune diseases located here, which needs to be further clarified.

MtDNA copy number changes could initiate diseases by decreasing the levels of mitochondrial respiratory chain enzyme complex, it elevated in a variety of diseases including cancer, cardiovascular diseases, neurodegenerative diseases, and rheumatism (Blokhin et al., 2008; Gu et al., 2013; Guo et al., 2013; Fang et al., 2014; Eirin et al., 2016). In line with this report, we found that mtDNA copy number was higher in SS patients. MtDNA spilled from cells could act as damage-related molecular patterns (DAMP) to activate pattern recognition receptors (PRRs) thereby to damage salivary gland cells by inducing inflammatory responses (Barrera et al., 2021). The elevated mtDNA copy number in SS patients may contribute to the accumulation of salivary gland cell damage, but the true mechanism remains further study.

We found that SS-risk SNPs were linked to elevated IL-2 and decreased IL-10 levels. MtDNA could both improve the production of IL-2 via the mitochondrial respiratory complex I-mediated oxidative signaling pathway and enhance the expression of IL-10 (Kaminski et al., 2010; Field et al., 2020). IL-2 may enhance the autoimmune response of SS patients and lead to the ultimate destruction of target organs (Ohyama et al., 1996; Moriyama et al., 2012). IL-10 is released by various regulatory cells to maintain immune tolerance (Pot et al., 2011; Brockmann et al., 2017). In people and animal models with SS, IL-10-producing regulatory B (Breg) cells inhibited T follicular helper (Tfh) cell-mediated SS development and its amount was negatively correlated with disease activity (Lin et al., 2019). All of above supports our finding that SS risk related D-loop SNPs might modify the SS by mediating cytokine expression. There is every chance that other genetic variants from nuclear genome or mitochondrial mediate the cytokine expression.

We couldn’t find the association for SS risk SNPs in the mitochondrial D-loop and ROS generation from blood sample of patient, but that doesn’t mean there is no association for these SNPs and mitochondrial ROS from targeted organs of SS patients. Further research is needed to uncover the link between mtDNA SNPs and ROS production in SS.

Our findings suggest that mitochondrial D-loop SNPs are predictors for SS risk, it might modify the SS development by regulating cytokine expression.

## Data Availability

The original contributions presented in the study are included in the article/Supplementary Material, further inquiries can be directed to the corresponding author/s.
