# Supplementary figures and images for: Mitochondrial Displacement Loop Region SNPs Modify Sjögren’s Syndrome Development by Regulating Cytokines Expression in Female Patients
Source: Front Genet. 2022 Mar 11;13:847521. doi: 10.3389/fgene.2022.847521 (PMC8963357; doi:10.3389/fgene.2022.847521)

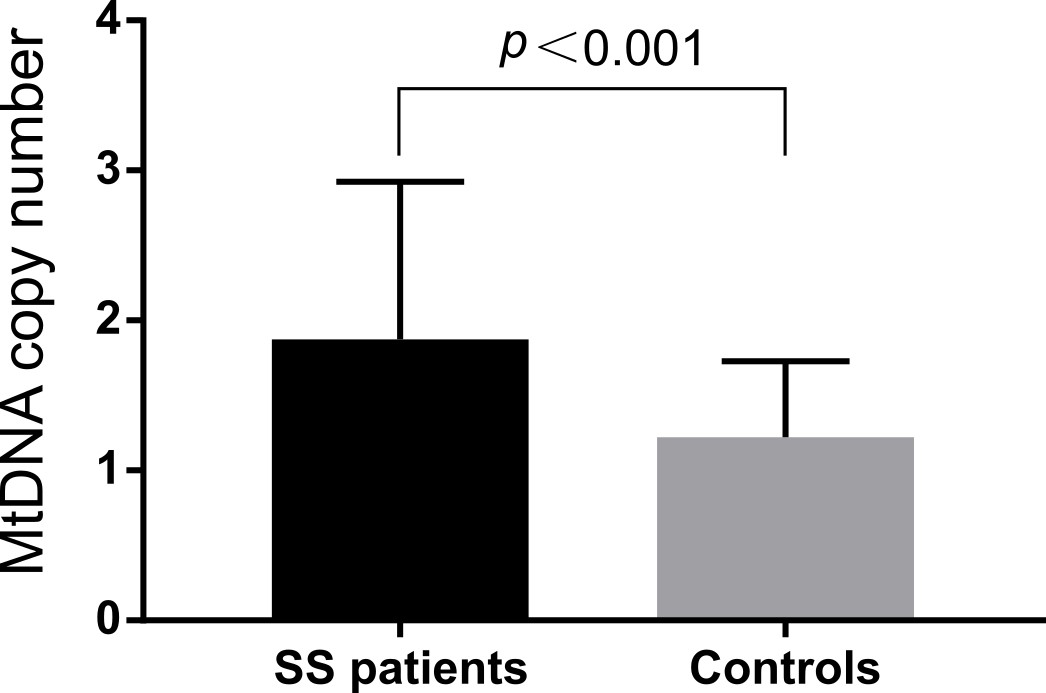

Supplement: Supplementary file 1 [file Image1.JPEG]

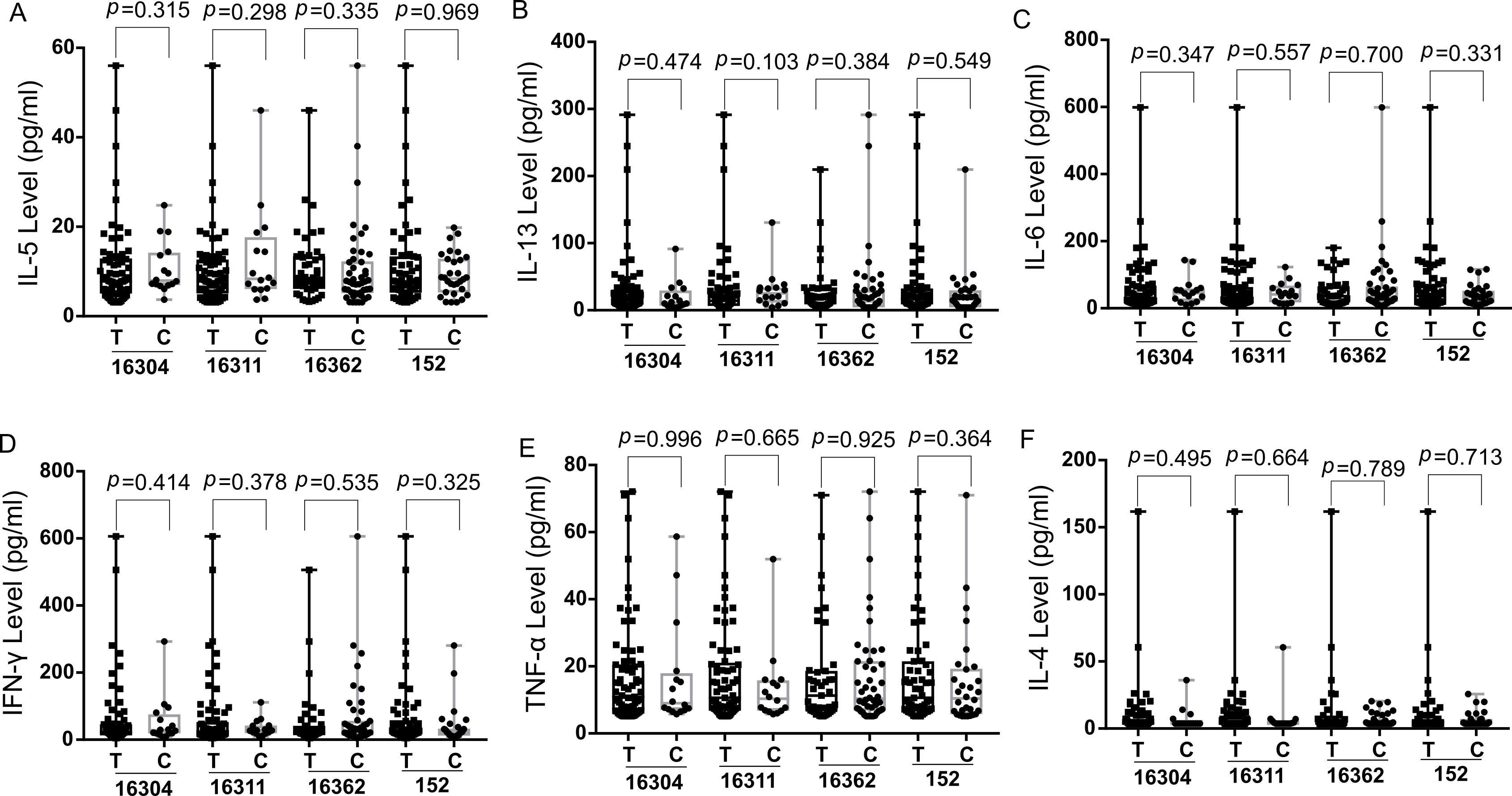

Supplement: Supplementary file 2 [file Image2.JPEG]
