# Supplementary material for: Mitochondrial Displacement Loop Region SNPs Modify Sjögren’s Syndrome Development by Regulating Cytokines Expression in Female Patients
Source: Front Genet. 2022 Mar 11;13:847521. doi: 10.3389/fgene.2022.847521 (PMC8963357; doi:10.3389/fgene.2022.847521)
Supplement: Supplementary file 3 [file DataSheet1.docx]

| **Table S1\|** Primers used in copy number analysis. | |
| --- | --- |
| HGB forward | 5' GTGCACCTGACTCCTGAGGAGA 3' |
| HGB reverse | 5' CCTTGATACCAACCTGCCCAG 3' |
| ND1 forward | 5'-CCCTAAAACCCGCCACATCT-3' |
| ND1 reverse | 5'-GAGCGATGGTGAGAGCTAAGGT-3' |
|  | |

| **Table S2\|** SS risk-associated single nucleotide polymorphism sites showing  frequency difference between SS patients and controls in an independent cohort. | | | | | |
| --- | --- | --- | --- | --- | --- |
|  |  |  |  |  |  |
| Nucleotide | SS  patients  (n=40) | Controls  (n=98) | χ² | *p* | OR |
|  |  |  |  |  |  |
|  |  |  |  |  |  |
| 16304T/C | 31/9 (77.5%/22.5%) | 90/8 (91.8%/8.2%) | 4.160 | 0.041 | 0.306 |
| 16311T/C | 31/9 (77.5%/22.5%) | 90/8 (91.8%/8.2%) | 4.161 | 0.041 | 0.306 |
| 16362T/C | 18/22 (44.5%/55.5%) | 63/35 (64.3%/35.7%) | 4.358 | 0.037 | 0.455 |
| 152T/C | 25/15 (62.5%/37.5%) | 78/20 (79.6%/20.4%) | 4.384 | 0.036 | 0.427 |
| SS: Sjogren's syndrome; χ²:Chi-square; *p*: probability value; OR: Odds ratio. | | | | | |
